# Identifying Sex of Neonate Turtles with Temperature-dependent Sex Determination via Small Blood Samples

**DOI:** 10.1038/s41598-020-61984-2

**Published:** 2020-03-19

**Authors:** Boris Tezak, Itzel Sifuentes-Romero, Sarah Milton, Jeanette Wyneken

**Affiliations:** 1 Department of Biological Sciences, Florida Atlantic University, Boca Raton, FL 33431-0991 USA; 2 Department of Cell Biology, Duke University Medical Center, Durham, NC 27710 USA

**Keywords:** Differentiation, Herpetology, Conservation biology

## Abstract

Temperature-dependent sex determination, present in most turtle species, is a mechanism that uses temperature to direct the sex of the embryo. The rapid increase of global temperatures highlights the need for a clear assessment of how sex ratios of organisms with TSD are affected. In turtles with TSD, quantifying primary sex ratios is challenging because they lack external dimorphism and heteromorphic sex chromosomes. Here we describe a new technique used to identify sex in neonate turtles of two TSD species, a freshwater turtle (*Trachemys scripta*) and a marine turtle (*Caretta caretta*) via analysis of small blood samples. We used an immunoassay approach to test samples for the presence of several proteins known to play an important role in sex differentiation. Our results show that Anti-Mullerian Hormone (AMH) can be reliably detected in blood samples from neonate male turtles but not females and can be used as a sex-specific marker. Verification of sex via histology or laparoscopy revealed that this method was 100% reliable for identifying sex in both *T. scripta* and *C. caretta* 1–2 day-old hatchlings and 90% reliable for identifying sex in 83–177 day-old (120–160 g) loggerhead juveniles. The method described here is minimally invasive, and for the first time, greatly enhances our ability to measure neonate turtle sex ratios at population levels across nesting sites worldwide, a crucial step in assessing the impact of climate change on imperiled turtle species.

## Introduction

Environmentally-dependent sex determining systems (ESD) are common in vertebrate animals^1^, but the developmental mechanisms involved remain poorly understood. When temperature is the critical factor for sex determination, the process is termed temperature-dependent sex determination (TSD)^2^. Many reptiles (including crocodilians, tuataras, some lizard taxa, and most turtles) lack sex chromosomes and instead have TSD. Under TSD, gonads differentiate into ovaries or testes depending on the incubation temperature of the eggs during a critical period of embryonic development^2^. In turtles, the most common pattern of TSD shows cool temperatures producing males and high temperatures yielding females^3–7^. Previous literature highlights the potential impacts of climate change (both via warmer averages and increased thermal fluctuations) on species with temperature-dependent sex determination^8,9^. With regards to most turtles, including all sea turtle species, the most common prediction is increased extinction risk associated with extremely female-biased sex ratios^9–12^. These concerns highlight the importance of identifying naturally occurring hatchling sex ratios in turtle species with TSD and using long-term data to predict how sex ratios will change under the predicted incubation conditions associated with global warming.

Identifying natural turtle hatchling sex ratios at nesting sites is challenging for a variety of reasons, spanning morphological limitations to ethical issues to a lack of understanding of the mechanisms that actually direct embryonic sexual differentiation^13^. Generally, turtles are characterized by being long lived, late maturing, and are not sexually dimorphic until approaching sexual maturity (e.g., marine turtle species often take over 15 years to become sexually mature)^1,14,15^. Due to the difficulties associated with readily identifying hatchling sex, most large scale studies investigating turtle hatchling sex ratios rely on nest temperatures, air temperatures^16–19^, and/ or nest incubation durations^20,21^ to estimate sex ratios indirectly. However, these proxies often fail to match the primary sex ratios from natural turtle nests or rookeries^8,16,22^. For instance, sample sex ratios collected from loggerhead sea turtles (*Caretta caretta)* in southeastern Florida reveal significant variability, with females being produced over a wider range of temperatures than those found in many well-controlled laboratory studies^23–25^. These inconsistencies expose the need for a reliable technique to accurately identify the sex of neonate turtles.

To date, a limited number of methods have been reported to reliably identify sex in neonate turtles with TSD. Valenzuela *et al*., (2004) describes a landmark-based geometric morphometric method to detect subtle sexual dimorphism in hatchlings of two freshwater turtle species which were incubated under constant temperatures^26^; this approach has since been used in other freshwater turtle species^27–30^. Another method requires sacrificing turtles to observe the histology of the gonads^18,31–34^, a destructive approach that limits the geographic range as well as the sample sizes because of ethical concerns (i.e., the imperiled status of many turtles)^4,13,35,36^. The third method is through laparoscopic examination of gonadal and accessory duct morphology^13,37,38^. This technique is invasive, requires specialized training, and may be impractical for large-scale studies as the turtles must be raised for several months until they reach adequate size and mass for safe laparoscopy. Radioimmunoassay analysis of estradiol-17β and testosterone ratios from chorioallantoic/amniotic fluid (CAF)^39,40^ has also been used to identify the sex of turtle hatchlings. However, this method requires extensive handling of the eggs prior to hatching in order to prevent contamination and ensure that enough CAF can be obtained for the analysis, making this approach logistically difficult and impractical for large-scale studies.

The sex of juvenile turtles (>1 year old) is often identified via blood hormone analyses^3,15,41–44^. Although this method has previously been used to sex neonates in some TSD turtle species^45,46^, this approach is seldom used because the target hormones occur in concentrations too low to discriminate sex in neonates of some turtle species^13^, and radioimmunoassay often requires blood volumes that may be too large (>100 uL) to obtain from neonates of small turtle species^13,15,42,43,47^.

**Figure 1:**
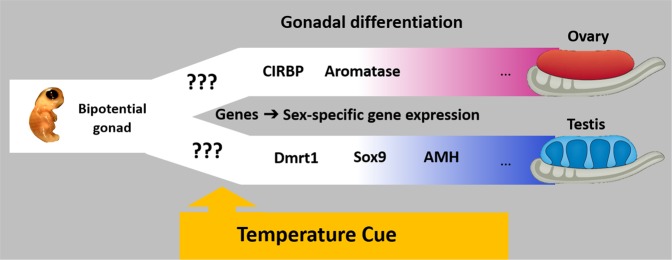
Diagram of relevant genetic events underlying gonad differentiation in reptiles at male and female promoting temperature^49,50^.

The shortcomings inherent in these methods inspired us to look for an alternative approach that could be used to identify sex in neonate turtles with TSD. We used previously published data from studies on the molecular mechanisms of sex determination and differentiation in vertebrates^48,49^ to identify a list of candidate proteins that could potentially be detected in blood samples to serve as sex specific markers of hatchling sex. To date, the exact mechanism of sex determination in organisms with TSD remains unresolved. However, downstream of the environmental sex determining trigger there is a well-studied network of molecular interactions and cellular responses that lead to the formation of the ovary or testis^48,49^ (Fig. 1).

Studies to unravel the developmental pathway of TSD in several reptile species have identified a number of genes that differ in expression depending upon male and female promoting temperatures (MPT and FPT, respectively)^48,49^. For instance at MPT, differentiating gonads express higher levels of Doublesex and mab-3 related transcription factor 1 (DMRT1), Sox9, and Anti-Müllerian hormone (AMH), while at FPT differentiating gonads upregulate Cold-inducible RNA-binding protein (*CIRBP)* and *CYP19A*1 (Aromatase) expression^48,50,51^. These contrasting profiles of sex-specific proteins were previously identified to direct sexual differentiation in developing gonads^37,50–53^. However, to our knowledge, differences in sex specific protein expression patterns have never been identified in blood samples of hatchlings with TSD.

Here we examined 1–2 day-old hatchling red-eared slider (*Trachemys scripta elegans)* and loggerhead *(Caretta caretta)* turtles to determine if sex specific proteins (Dmrt1, Sox9, Amh, Aromatase and CIRBP) could be detected in blood samples via Western Blot analysis. We then verified the accuracy of this sex identification approach via histology or laparoscopic examination. Finally, we tested the applicability of the new method to identify the sex of 83–177 day-old (120–160 g) loggerhead juveniles. For species with TSD, the risk of sex ratios becoming dangerously skewed increases as global temperatures continue to rise^10–12^. Our results provide the field of reptile conservation, and particularly turtle conservation and management with a valuable tool that can be used to accurately assess the sex ratios of hatchlings at the population level in turtle nesting sites worldwide. This information could be critical when developing conservation measures for species with TSD.

## Results and Discussion

**Figure 2:**
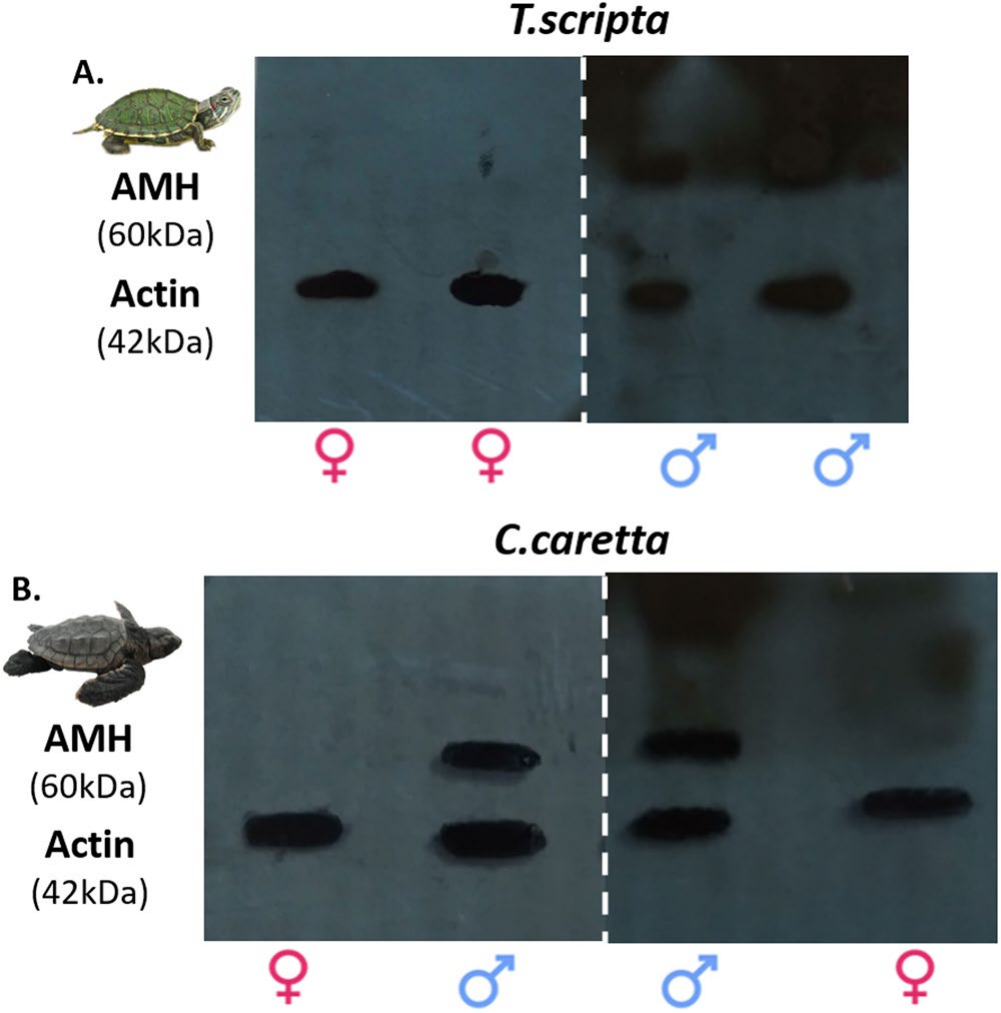
AMH Western Blots of male and female *T. scripta* (**A**) and *C. caretta* (**B**) hatchling (1–2 days old) blood samples. Dashed white line indicates separate blots. Actin (42 kDa) was used as our loading control and was present in all samples analyzed. Presence of AMH (~60 kDa) is easily detected in male samples but it is absent in female samples.

Due to the imperiled status and strict regulations associated with sampling loggerhead turtles, we first tested the immunoassay approach to identify sex on an abundant species, the red-eared slider (*T. scripta*). Hatchling samples incubated at constant male producing temperatures (MPT) and female producing temperature (FPT) in the lab to ensure the production of both males and females. Western blot (WB) immunoassays on ten gonad samples verified that the commercially available antibodies adequately identify the presence of the proteins of interest in red-eared slider turtle samples (Table 1). All 20 hatchling blood samples (collected within the first 2 days post hatching) analyzed via WB tested negative for Sox9, Dmrt1, CIRBP and Aromatase, whereas 10 samples tested positive for AMH (Fig. 2A; Tables 2 and S1 in supplement). The sex of each turtle was verified independently using Hematoxylin and Eosin (H&E) stained histological sections, based on the organization in the medullary region and thickness of the cortical layer in the gonad^13,37^. These results confirmed that AMH is released into the bloodstream of *T. scripta* male hatchlings and provided enough support for us to test the same sex identification approach in *C. caretta* hatchlings.

**Table 1:** Summary of Western Blot results from red-eared slider and loggerhead gonad samples.

	Incubation Treatment	Sox9	Dmrt1	AMH	CIRBP	Aromatase	Males	Females
*T. scripta*	MPT (N = 5)	5/5	5/5	5/5	5/5	0/5	5	0
	FPT (N = 5)	0/5	0/5	0/5	5/5	5/5	0	5
*C.caretta*	MPT (N = 2)	2/2	2/2	2/2	2/2	0/2	2	0
	FPT (N = 3)	0/3	0/3	0/2	3/3	3/3	0	3

Sex was verified independently via H&E histological sections. All gonads were collected from 1–2 day old hatchlings. Note that CIRBP was detected in gonad samples from both male and female hatchlings. The numerators indicate how many of the individuals tested were positive for the protein.

**Table 2:** Summary of Western Blot results from red-eared slider and loggerhead hatchling (1–2 days old) blood samples.

	Incubation Treatment	Sox9	Dmrt1	AMH	CIRBP	Aromatase	Males	Females	WB vs. Laparoscopy
*T. scripta*	MPT (N = 10)	0/10	0/10	10/10	0/10	0/10	10	0	100%
	FPT (N = 10)	0/10	0/10	0/10	0/10	0/10	0	10	100%
*C.caretta*	MPT (N = 28)	0/15	0/15	23/28	0/0	0/15	23	5	100%
	FPT (N = 31)	0/15	0/15	0/31	0/0	0/15	0	31	100%

Columns labeled “Males” and “Females” display the number of turtles per treatment which were identified as male or female via gonad histology (red-eared sliders) or laparoscopic examination of gonad and gonadal ducts (loggerheads).

WB immunoassays on two gonad samples from *C. caretta* turtles incubated at 27.5 °C (a temperature that produced 82% males, that is, 23 males out of 28 total hatchlings) detected Sox9, Dmrt1, CIRBP, and AMH and did not detect Aromatase. Three gonad samples from turtles incubated 32 °C (a temperature that produced 100% females; 31 females out of 31 total hatchlings) only tested positive for Aromatase and CIRBP (Table 1). The sex of each of the hatchlings found dead in the nest boxes was verified using H&E stained histological sections, based on the organization in the medullary region and thickness of the cortical layer in the gonad sections ^13,37^. The positive WB results obtained from the gonad samples indicated that the commercially available antibodies should identify the presence of the proteins of interest in loggerhead turtle samples as well. However, since CIRBP was found in both male and female gonads, we excluded it from further WB analyses of loggerhead blood samples as it would not serve as a sex specific marker and our plasma volume per sample was limited (~0.06 mL per hatchling sample).

Results from the 59 *C. caretta* hatchling plasma samples analyzed were consistent with the results obtained with the *T. scripta* plasma samples. We did not detect Sox9, Dmrt1, and Aromatase (Tables 2; S2 and S3 in supplement), showing that these proteins are not normally in bloodstream of loggerhead hatchlings. These results precluded the possibility of using these proteins as sex-specific markers to identify hatchling sex via analysis of blood samples. On the other hand, 23 of the 28 plasma samples from hatchlings incubated at ‘colder’ temperatures tested positive for Anti-Müllerian hormone (AMH) and later these 23 turtles were all verified as males via laparoscopy. All 31 hatchling plasma samples from the ‘warmer’ treatment tested negative for AMH and later those turtles were identified as females (Tables 2; S2 and S3 in supplement). The five plasma samples from the ‘colder’ treatment that tested negative for AMH were later identified as females via laparoscopy.

In sum, all samples positive for AMH came from male hatchlings, as verified independently via laparoscopic examination. The other 36 blood samples came from turtles identified as females (Tables S2 and S3 in supplement). Based on these results, we used the presence of AMH as the diagnostic trait to identify hatchlings as males; if the sample tested negative for AMH, it was classified as female (Fig. 2). The use of the AMH WB approach produced results that were 100% consistent with the results from the laparoscopic procedure. It is also important to highlight that the WB approach correctly identified the sex of five female hatchlings that were incubated at a temperature that produced mostly males, rejecting the possibility that our results are confounded by incubation temperature. The presence of AMH in hatchling turtle blood samples is consistent with previous studies on sexual development in mammals which show that AMH is secreted at high levels into the bloodstream until reaching sexual maturity^54^. Additionally, the male-specific expression of AMH can be explained by the fact that this hormone plays a critical role in male differentiation by controlling the degeneration of the paramesonephric (Müllerian) duct which would otherwise become the oviduct in females^55–58^.

After determining that presence of AMH in blood samples was a reliable approach to identify sex in loggerhead hatchlings, we conducted an initial assessment of the efficacy of this approach for identifying sex in juvenile loggerhead samples (Table 3). A second blood sample was collected from 20 of the turtles three days after the laparoscopic examination (at 83–177 days of age and 120–160 g), which included 10 males from 27.5 °C and 10 females from 32 °C. Eight out of these 20 juveniles tested positive for AMH, indicating that the agreement between the WB sex identification technique and the laparoscopic procedure decreased to 90% [(18/20); Tables 3; S4 in supplement]. That is, 18 out of 20 juvenile turtles were classified correctly while one juvenile female (177 days old) was incorrectly identified as a male and one juvenile male (159 days old) was misidentified as a female. Upon closer look at the video recordings from the laparoscopic procedure, we verified that the male that was incorrectly identified as a female was completely lacking any signs of a paramesonephric duct (PD). This observation suggests that AMH might not be a reliable sex marker for male turtles after full regression of the PD is complete. However, our results from the laparoscopic procedure show that in some cases, the PD is still partially present in some males even 8 months post-hatching. Moreover, remnant PD are found occasionally in necropsies of large juvenile and adult male loggerheads (Wyneken 2001). These observations are evidence that the time it takes for the PD to fully degenerate likely varies among individuals (and species), making it difficult to designate exactly after how long post-hatching this new method of identifying sex remains 100% accurate. Clearly, further investigation of the variation in PD regression and the use of AMH as a sex-specific predictor in older turtles is needed.

**Table 3:** Summary of Western Blot results from 83–177 day old loggerhead juvenile blood samples.

	AMH	Males	Females	WB vs. Laparoscopy
MPT (N = 10)	7/10	8	2	90%
FPT (N = 10)	1/10	0	10	90%

Note that the accuracy of the WB approach of sex identification decreased to 90%.

The female juvenile that was misidentified as a male via WB analysis had an unusual PD with an incomplete lumen. Note that Wyneken *et al*.^13^ reported that loggerhead female post-hatchlings normally have large mobile ducts, and females with an incomplete PD lumen are rare (nine out of 154 in that study). It is possible that the incomplete lumen in the post-hatchling female from this current study was caused by uncharacteristic expression of AMH, explaining our error when identifying sex via WB. Interestingly, in mammals, AMH has been reported to be produced by granulosa cells in the ovary throughout folliculogenesis. In fact, AMH has been reported to be a useful marker for identifying the quantity and quality of the ovarian follicle pool, and as a marker for both polycystic ovary syndrome and granulosa cell tumors in humans^59^. Although this has not been reported in any reptile species, it does provide a possible explanation for the unusual expression of AMH detected in our juvenile female sample.

The molecular signals orchestrating sexual differentiation are thought to be highly conserved across taxa^49,51,55,60^. Male specific expression of AMH in developing gonads is consistent even among different sex determining systems and has been reported in a number of vertebrate species ranging from mammals to fish^56,61–63^. Additionally, here we show that the same sex-specific pattern of AMH expression was found in hatchling blood samples from two turtle species that are phylogenetically and ecologically distinct. Together, these observations suggest that the WB approach for sex identification could potentially be used to reliably identify the sex in a number of other ESD species lacking sexual dimorphism.

Establishing primary sex ratios is a critical step when assessing the reproductive potential of a population. However, turtle hatchling sex ratios from nests that incubate in the field are rarely known^9,45,64–67^ and sex ratios inferred from laboratory temperature-sex ratio results alone can often be too simplistic or flawed^8,22,24^ to be informative of natural sex ratios. The results from our study strongly support the efficacy of AMH as a sex-specific marker that is reliably detected by immunoassay analysis (e.g., WB) in hatchling turtle blood samples. In the present study, the cost of the sample analysis was approximately ~$18.00 US per sample not including labor and equipment use. Current efforts from our lab are focusing on streamlining this technique; the ultimate goal is to develop a relatively inexpensive “field kit” to quickly and reliably identify hatchling sex in remote locations where access to a lab may be difficult.

In comparison to the current molecular methods for sex identification in turtles with TSD, the WB approach is quick, minimally invasive (requires small volume of blood), and the hatchling turtle can then be released immediately. We suggest that this novel technique will allow for more accurate estimates of hatchling sex ratios at a population level and on a global scale. That information should enable managers to precisely monitor changes in sex ratios that might arise as a consequence of changes in temperature over time, estimate how climate change will affect future generations of hatchlings, and expedite the evaluation of management strategies used to reduce alterations of sex ratios in turtles and other reptile species with temperature dependent sex determination.

## Materials and Methods

### Egg collection and incubation

#### Trachemys scripta elegans

Freshly laid red-eared slider (*T. scripta*) eggs from multiple clutches were obtained from Concordia Turtle Farms (Hammond, Louisiana USA) and transported by automobile in vacuum-sealed bags to our laboratory at Florida Atlantic University, Boca Raton, Florida USA. Fertilized eggs (determined by the presence of the white spot on the eggshell^68^) were randomly distributed in Styrofoam boxes (incubators) containing moist sterilized sand from a local beach and housed in incubators kept at 26 °C (male-promoting temperature or MPT) or 31 °C (female-promoting temperature or FPT)^69^. Temperatures in both chambers were controlled by an Omega iSeries Temperature & Process Controller Model CNi3233, (Norwalk, Connecticut USA; detailed in Lolavar and Wyneken 2017). Chamber temperature and air humidity were monitored every 30 min using HOBO U12 Temperature & Relative Humidity data loggers (accuracy ± 0.35 °C, resolution = 0.03 °C; Onset Corp., Bourne, Massachusetts, USA).

#### Caretta caretta

Due to sampling limitations associated with working with a protected species, the loggerhead hatchlings and post-hatchlings used in this study were also subjects of part of a separate concurrent study of incubation conditions (Lolavar & Wyneken, unpublished). Briefly, Loggerhead eggs were collected the morning following oviposition at Juno Beach, Florida USA. (26.88 N, 80.05 W) during the 2017 and 2018 nesting seasons. Eggs were transported by automobile in vacuum-sealed bags to our laboratory at Florida Atlantic University, located ~60 km (<45 min) away. Clutches were divided evenly into two groups of eggs and placed in Styrofoam boxes containing sterilized sand from a local beach. The boxes were then placed in two adjacent incubation chambers, one set at 27.5 °C (“cool”) and the other at 32 °C (“warm”). Although these incubation temperatures were expected to produce 100% males and 100% females, respectively^25^, we obtained 82% males at 27.5 °C and 100% females at 32 C°. Temperatures in both chambers were controlled and maintained as described above.

### Sample collection

#### Trachemys scripta elegans

Hatchling blood samples (~0.12 ml) were collected within two days of the hatching event. Blood samples were taken from the ventral caudal vein using heparinized syringes (Allergy Syringe with PrecisionGlide Needle [26 G ½ inch] Becton Dickinson, Franklin Lakes, New Jersey USA), immediately centrifuged, and the plasma was stored at – 80 °C for later processing. After blood collection, hatchlings were sacrificed via decapitation and gonad pairs were extracted, one of the gonads was used to verify the viability of the antibodies in *T. scripta* samples (stored at −80 °C) and the other gonad was fixed in 4% paraformaldehyde solution (PFA) and used to verify hatchling sex via H & E stained histological sections. A total of 20 *T. scripta* hatchlings were sampled for this study (10 from MPT and 10 from FPT).

#### Caretta caretta

When the eggs began to pip, they were placed individually into separately labeled containers so that hatchlings could be identified by their original clutch as well as by their experimental treatment. Once hatchlings internalized their yolk and had flattened plastrons, they were marked with an identification number denoting their incubation treatment, and transported in coolers ~15 min to the Florida Atlantic University Marine Laboratory where they were weighed before blood collection. A safe volume of blood based upon each turtle’s mass was collected on the same day that they were transported to lab. Blood volume (ml) in reptiles is about 5–8% of their total body weight. Mader & Rudloff (2006)^47^ suggests that 10% of that volume can be safely collected without harming the animal. For example, a 17 g hatchling has about ~1.3 ml of blood; a 10% sample of 0.13 ml would be safe. The minimum amount of plasma needed to perform western blots for our analyses is ~ 0.06 ml and the volume of plasma is roughly half of the blood volume.

Blood samples were taken from the external jugular vein using heparinized syringes (Allergy Syringe with PrecisionGlide Needle [26 G ½ inch] Becton Dickinson, Franklin Lakes, New Jersey USA), immediately centrifuged, and the plasma was stored at −80 °C for later processing. Alternatively, when access to a −80 °C was unavailable, plasma samples were stored in −20 °C freezer for up to two weeks and then moved to – 80 °C until processing. Plasma samples were labeled using a new ID code to prevent prior knowledge of the turtle’s sex (or incubation conditions) before performing the western blots. A total of 59 loggerhead hatchlings turtles were sampled throughout the course of the 2017 and 2018 nesting seasons (31 from the “warm” incubator and 28 from the “cool” incubator). In addition, gonad samples were collected from hatchlings found dead in nest boxes (less than 24 h following death) to verify the viability of the antibodies in loggerhead turtle samples. A total of 5 gonad pairs were harvested (three from the “warm” and two from “cool” incubators) from the dead turtles; one of the gonads was stored at −80 °C for protein extraction while the corresponding gonad was fixed in 4% PFA for sex verification via histology.

Hatchlings were then raised until they reached a minimum of 120 g and sex identified via laparoscopic examination of the gonads and gonadal ducts following the protocol described in Wyneken *et al*.^13^. After laparoscopy, the turtles were allowed to recover in the lab for three days. Blood samples were taken from a subset of those turtles (10 from the “cool” and 10 from the “warm” incubators), following the same blood collection procedure described for the hatchlings. The second set of blood samples was taken to test if the “sex specific” proteins remained present and detectable in 83–177 day-old (120–160 g) juveniles. All turtles were then released offshore in the Florida Gulfstream Current minimally one week following laparoscopy.

#### Protein extractions and western blots

Gonad samples from both *T. scripta* and *C. caretta* hatchlings were used to ensure the specificity and viability of the antibodies used. Frozen tissues were thawed for ~15 min and proteins were extracted in RIPA buffer (1 M NaCl, 0.5 M EDTA, 1% Triton X100, 0.5 M Tris-Cl pH 7.4; with added 5 M DDT, 0.1 M PMSF, 5 M mercaptoethanol, 3 mM protease inhibitor (PI) diluted 1:1000) using a glass homogenizer at 22 °C. The homogenate was centrifuged at 15,000 rpm at 4 °C for 10 min and the supernatant was collected for further analysis. Protein concentrations in each sample (both gonad and plasma) were determined using a standard Bicinchoninic Acid Assay (BCA) following manufacturer’s protocol (Pierce Biotechnology, Inc., Rockville, Illinois, USA).

AMH (1:200), Aromatase (1:500), Sox9 (1:1000), CIRBP (1:500), and Dmrt1 primary antibodies [(1:1000); all from Aviva Systems Biology, San Diego, California USA] were diluted in 5% non-fat milk in Tris Buffered Saline with Tween (TBST; 25 mM Tris-Cl pH 7.5, 150 mM NaCl, 1% Tween 20). The primary antibody against β-actin (Cell Signaling Technology, Davers, Massachusetts, USA) diluted at 1:3000, was used as loading controls in 5% non-fat dried milk in TSBT. Secondary anti-rabbit antibody (Enzo Life Sciences Inc., Farmingdale, NY) was diluted 1:4000 and secondary anti-mouse antibody was diluted 1:3000 (Boster Biological Technology, Pleasanton, California, USA). Equal amounts of protein (30 µg/ml) were separated in a 12% SDS Polyacrylamide gel for 1 h at 150 V. The proteins were transferred onto a nitrocellulose membrane (Amersham Biosciences, Pittsburgh, Pennsylvania, USA) for 1 h at 0.3 A. The membrane was blocked in 5% non-fat dried milk in TBST for 1 h at room temperature. Membranes were incubated with the primary antibody and then rocked gently overnight at 4 °C. The membranes were washed 3 × 5 min in TBST and probed with corresponding secondary antibody for 1 h at room temperature. Immunoactive protein bands were detected with enhanced chemiluminescence (ECL) (Thermo-Fisher Scientific, Grand Island, New York, USA). Blots were developed on x-ray film and pictures were then taken using a digital camera. No additional picture processing was performed. *T. scripta* samples were processed first in order to assure the efficacy of this approach before sampling an imperiled marine turtle species. *C. caretta* samples were then processed in random order throughout the course of a six-month period post sample collection.

All work was done in accordance with animal care and use protocols approved by the Institutional Animal Care and Usage Committee at Florida Atlantic University (IACUC A16-19). Blood sample collection and laparoscopic examination were permitted through Florida Fish and Wildlife Conservation Commission (FWC Permits: MTP# 073 A and MPT# 216).

## Supplementary information

Supplementary Information.

## Data Availability

The datasets generated during and/or analyzed during the current study are available from the corresponding author on reasonable request.
