# Supplementary material for: Identifying Sex of Neonate Turtles with Temperature-dependent Sex Determination via Small Blood Samples
Source: Sci Rep. 2020 Mar 19;10:5012. doi: 10.1038/s41598-020-61984-2 (PMC7081227; doi:10.1038/s41598-020-61984-2)
Supplement: Supplementary file 1 — Supplementary Information. [file 41598_2020_61984_MOESM1_ESM.docx]

**Title:** Identifying Sex of Neonate Turtles with Temperature-dependent Sex Determination via Small Blood Samples

**Authors:** Boris Tezak^1, 2*^, Itzel Sifuentes-Romero^1^, Sarah Milton^1^, Jeanette Wyneken^1^

1. Department of Biological Sciences, Florida Atlantic University, Boca Raton, FL 33431-0991 USA

2. Department of Cell Biology, Duke University Medical Center, Durham, NC 27710, USA

***Corresponding author**: Boris Tezak, Department of Biological Sciences, Florida Atlantic University, 777 Glades Rd, Boca Raton, FL 33431-0991, USA. [btezak@fau.edu](mailto:btezak@fau.edu)

**SUPPLEMENTARY INFORMATION**

**Table S.1**. Western Blot results of blood samples from 1-2 days old red-eared slider (*Trachemys scripta*) hatchlings incubated at female-promoting temperatures and male-promoting temperatures (“FPT” and “MPT, respectively). “X” designates no protein presence detected via WB while “**+**” designates protein detection. Sex was verified via histological examination of the gonads.

| **Incubation Treatment** | **ID** | **Sox9** | **Dmrt1** | **AMH** | **Aromatase** | **Sex (Histology)** |
| --- | --- | --- | --- | --- | --- | --- |
| FPT | Ts_1 | X | X | X | X | **F** |
| FPT | Ts_2 | X | X | X | X | **F** |
| FPT | TS_3 | X | X | X | X | **F** |
| FPT | Ts_4 | X | X | X | X | **F** |
| FPT | Ts_5 | X | X | X | X | **F** |
| FPT | Ts_6 | X | X | X | X | **F** |
| FPT | Ts_7 | X | X | X | X | **F** |
| FPT | Ts_8 | X | X | X | X | **F** |
| FPT | Ts_9 | X | X | X | X | **F** |
| FPT | Ts_10 | X | X | X | X | **F** |
| MPT | Ts_11 | X | X | **+** | X | **M** |
| MPT | Ts_12 | X | X | **+** | X | **M** |
| MPT | Ts_13 | X | X | **+** | X | **M** |
| MPT | Ts_14 | X | X | **+** | X | **M** |
| MPT | Ts_15 | X | X | **+** | X | **M** |
| MPT | Ts_16 | X | X | **+** | X | **M** |
| MPT | Ts_17 | X | X | **+** | X | **M** |
| MPT | Ts_18 | X | X | **+** | X | **M** |
| MPT | Ts_19 | X | X | **+** | X | **M** |
| MPT | Ts_20 | X | X | **+** | X | **M** |

**Table S.2.** Western Blot results of blood samples from 1-2 days old loggerhead (*Caretta caretta*) hatchlings incubated at “warmer” (female biasing; 32 °C) temperature. “X” designates no protein presence detected via WB; blank boxes indicate that the sample was not tested for that particular protein. Sex was verified by laparoscopic exams.

| **Gumbo Limbo ID** | **WB ID** | **Sox9** | **Dmrt1** | **AMH** | **Aromatase** | **Sex (Laparoscopy)** |
| --- | --- | --- | --- | --- | --- | --- |
| AY 101 | WB_XM | X | X | X | X | **F** |
| AY 102 | WB_FF |  |  | X |  | **F** |
| AY 108 | WB_RP |  |  | X |  | **F** |
| AY 109 | WB_PT | X | X | X | X | **F** |
| AY 110 | WB_SS | X | X | X | X | **F** |
| AY 201 | WB_CT | X | X | X | X | **F** |
| AY 202 | WB_TL |  |  | X |  | **F** |
| AY 209 | WB_MY | X | X | X | X | **F** |
| AY 210 | WB_KK | X | X | X | X | **F** |
| AY 301 | WB_HP |  |  | X |  | **F** |
| AY 304 | WB_RR |  |  | X |  | **F** |
| AZ 104 | WB_AS | X | X | X | X | **F** |
| AZ 109 | WB_QQ | X | X | X | X | **F** |
| AZ 204 | WB_QP | X | X | X | X | **F** |
| AZ 209 | WB_TI |  |  | X |  | **F** |
| AZ 210 | WB_BL |  |  | X |  | **F** |
| AZ 301 | WB_RX |  |  | X |  | **F** |
| AZ 302 | WB_KZ | X | X | X | X | **F** |
| AZ 409 | WB_ZT |  |  | X |  | **F** |
| AZ 410 | WB_ZA | X | X | X | X | **F** |
| AZ 508 | WB_DL |  |  | X |  | **F** |
| AZ 601 | WB_DD |  |  | X |  | **F** |
| AZ 602 | WB_BH | X | X | X | X | **F** |
| AZ 609 | WB_FX |  |  | X |  | **F** |
| AZ 610 | WB_YA | X | X | X | X | **F** |
| AZ 701 | WB_PL | X | X | X | X | **F** |
| AZ 702 | WB_PB |  |  | X |  | **F** |
| AZ 704 | WB_PQ |  |  | X |  | **F** |
| AZ 810 | WB_UU |  |  | X |  | **F** |
| AZ 901 | WB_UA |  |  | X |  | **F** |
| AZ 904 | WB_ZC | X | X | X | X | **F** |

**Table S.3.** Western Blot results of blood samples from 1-2 days old loggerhead (*C.caretta*) hatchlings incubated at “cooler” (male biasing; 27.5 °C) temperatures. “X” designates no protein presence detected via WB while “**+**” designates protein detection; blank boxes indicate that the sample was not tested for that particular protein. Sex was verified by laparoscopic exams.

| **Gumbo Limbo ID** | **WB ID** | **Sox9** | **Dmrt1** | **AMH** | **Aromatase** | **Sex (Laparoscopy)** |
| --- | --- | --- | --- | --- | --- | --- |
| AA 102 | WB_AA |  |  | **+** |  | **M** |
| AA 104 | WB_AB |  |  | **+** |  | **M** |
| AA 106 | WB_BB | X | X | **+** | X | **M** |
| AA 204 | WB_ZZ | X | X | **+** | X | **M** |
| AA 205 | WB_XB |  |  | **+** |  | **M** |
| AA 208 | WB_AD | X | X | **+** | X | **M** |
| AA 304 | WB_FA |  |  | **+** |  | **M** |
| AA 305 | WB_CC |  |  | **+** |  | **M** |
| AA 307 | WB_BF | X | X | **+** | X | **M** |
| AA 310 | WB_SD |  |  | **+** |  | **M** |
| AA 507 | WB_MM |  |  | **+** |  | **M** |
| AA 508 | WB_MN | X | X | **+** | X | **M** |
| AA 510 | WB_KP | X | X | **+** | X | **M** |
| AA 601 | WB_KY | X | X | **+** | X | **M** |
| AA 602 | WB_BC |  |  | **+** |  | **M** |
| AA 606 | WB_HH | X | X | **+** | X | **M** |
| AA 608 | WB_RB | X | X | **+** | X | **M** |
| AA 710 | WB_RC | X | X | **+** | X | **M** |
| AA 801 | WB_GG |  |  | **+** |  | **M** |
| AA 802 | WB_GF |  |  | **+** |  | **M** |
| AA 808 | WB_PP |  |  | **+** |  | **M** |
| AA 809 | WB_BT | X | X | **+** | X | **M** |
| AA 810 | WB_NN | X | X | **+** | X | **M** |
| AA 306 | WB_LL | X | X | X | X | **F** |
| AA 308 | WB_WX |  |  | X |  | **F** |
| AA 401 | WB_XC | X | X | X | X | **F** |
| AA 605 | WB_MO | X | X | X | X | **F** |
| AA 607 | WB_HB |  |  | X |  | **F** |

**Table S.4**. Results from Western Blots and laparoscopies on loggerhead (*C.caretta*) juveniles. “Age” column designates the days post hatching for each individual turtle at the time of the second blood draw. Note, second set of blood draws were always performed three days after laparoscopy. “X” designates no protein presence detected via WB while “**+**” designates protein detection; gray highlighted rows show the turtles for which sex identification via WB did not match the sex verified via laparoscopy.

| **Gumbo Limbo ID** | **WB ID** | **AMH** | **Sex (Laparoscopy)** | **Age**  **(Days)** |
| --- | --- | --- | --- | --- |
| AY 101 | WB_XM | X | **F** | **122** |
| AY 102 | WB_FF | X | **F** | **122** |
| AY 201 | WB_CT | X | **F** | **94** |
| AY 304 | WB_RR | X | **F** | **144** |
| AZ 409 | WB_ZT | X | **F** | **108** |
| AZ 410 | WB_ZA | X | **F** | **108** |
| AZ 602 | WB_BH | X | **F** | **177** |
| AZ 601 | WB_DD | + | **F** | **177** |
| AZ 702 | WB_PB | X | **F** | **170** |
| AZ 901 | WB_UA | X | **F** | **165** |
| AA 401 | WB_XC | X | **F** | **124** |
| AA 605 | WB_MO | X | **F** | **151** |
| AA 606 | WB_HH | **+** | **M** | **83** |
| AA 710 | WB_RC | **+** | **M** | **123** |
| AA 808 | WB_PP | X | **M** | **159** |
| AA 102 | WB_AA | **+** | **M** | **173** |
| AA 106 | WB_BB | **+** | **M** | **152** |
| AA 305 | WB_CC | **+** | **M** | **120** |
| AA 507 | WB_MM | **+** | **M** | **151** |
| AA 508 | WB_MN | **+** | **M** | **127** |


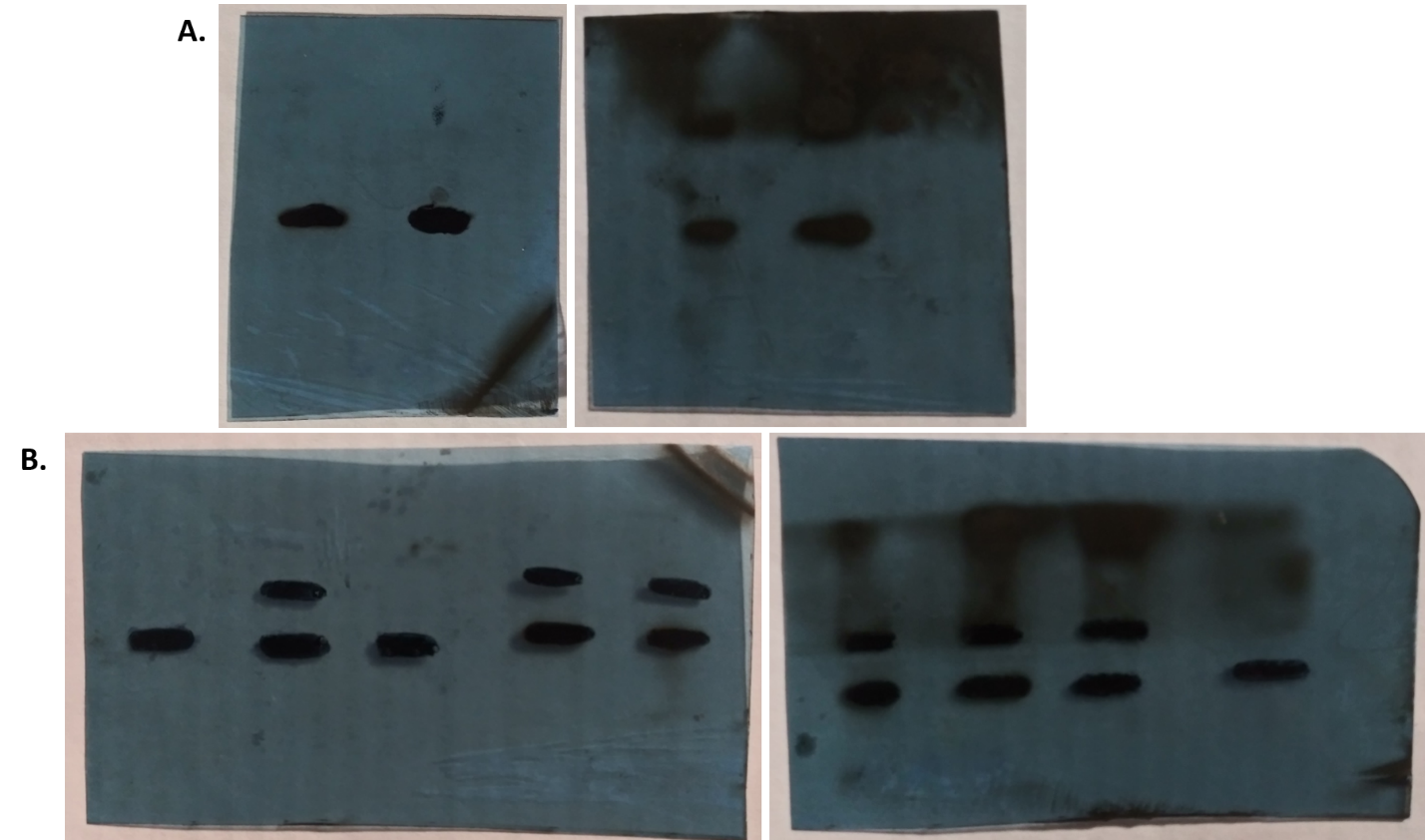


**Figure S.1.** Original, unprocessed images of AMH Western Blots of *T.scripta* (A) and *C.caretta* (B) hatchling (1-2 days old) blood samples**.** Actin (42kDa) was used as our loading control and was present in all samples analyzed. Presence of AMH (~ 60 kDa) is easily detected in male samples but it is absent in female samples.
